# Photoplethysmographic Measurement of Arterial Stiffness in Polish Patients with Long-COVID-19 Syndrome—The Results of a Cross-Sectional Study

**DOI:** 10.3390/diagnostics12123189

**Published:** 2022-12-16

**Authors:** Izabela Szoltysek-Boldys, Wioleta Zielinska-Danch, Danuta Loboda, Jacek Wilczek, Michal Gibinski, Elzbieta Paradowska-Nowakowska, Krzysztof S. Golba, Beata Sarecka-Hujar

**Affiliations:** 1 Department of General and Inorganic Chemistry, Faculty of Pharmaceutical Sciences in Sosnowiec, Medical University of Silesia in Katowice, 41-200 Sosnowiec, Poland; 2 Department of Electrocardiology and Heart Failure, Medical University of Silesia in Katowice, 40-635 Katowice, Poland; 3 Department of Electrocardiology, Upper-Silesian Medical Centre in Katowice, 40-635 Katowice, Poland; 4 Department of Cardiac Rehabilitation, “Ustron” Health Resort, 43-450 Ustron, Poland; 5 Department of Basic Biomedical Science, Faculty of Pharmaceutical Sciences in Sosnowiec, Medical University of Silesia in Katowice, 41-200 Sosnowiec, Poland

**Keywords:** arterial stiffness, COVID-19 disease, SARS-CoV-2, cardiovascular risk

## Abstract

The coronavirus disease 2019 (COVID-19) is associated with an increase in the incidence of cardiovascular diseases (CVD) that persists even several months after the onset of infection. COVID-19 may also have an impact on arterial stiffness, which is a risk factor for CVD. We aimed to analyze if and to what extent arterial stiffness measured by photoplethysmography differed among COVID-19 convalescents depending on the acute phase severity and time elapsed since disease onset. A total of 225 patients (mean age 58.98 ± 8.57 years, 54.7% women) were analyzed after COVID-19 hospitalization at the Cardiac Rehabilitation Department of the Ustron Health Resort (Poland). In the entire study population, no differences were found in the mean values of stiffness index (SI) and reflection index (RI) depending on the severity of the acute COVID-19 and the time since the onset of the disease. There were no differences in the heart rate (HR) according to the severity of acute COVID-19; the mean HR was higher in patients who had COVID-19 less than 12 weeks before the study than in convalescents more than 24 weeks after the acute disease (*p* = 0.002). The mean values of SI and RI were higher in men than in women (*p* < 0.001), while the heart rate (HR) was similar in both sexes (*p* = 0.286). However, multiple linear regression analyses after adjusting for factors influencing arterial stiffness, i.e., sex, age, body mass index, smoking status, hypertension, diabetes, the severity of the acute COVID-19, and the time from the disease onset, confirmed that age, sex, time from disease onset, and diabetes are the most important determinants that could influence arterial stiffness.

## 1. Introduction

The coronavirus disease-2019 (COVID-19), caused by Severe Acute Respiratory Syndrome Coronavirus-2 (SARS-CoV-2), leading to severe interstitial pneumonia, has been troubling people around the world since 2019 [1]. By November 2022, 629 million cases and 6.5 million deaths due to COVID-19 have been confirmed worldwide [2]. Surviving COVID-19 resulted in a deterioration of the general fitness, quality of life, and cardio-respiratory capacity of a large number of convalescents [3,4,5].

It was demonstrated that cardiovascular diseases (CVD) and the presence of cardiovascular (CV) risk factors, including older age, hypertension, diabetes, dyslipidemia, obesity, and smoking, significantly increase the risk of developing severe COVID-19 [6,7,8]. However, studies conducted on patients recovering from COVID-19 prove that the disease is also associated with an increase in the incidence of newly diagnosed CVD and diabetes, and the increase in risk persists for up to several months and is independent of age, sex, and other traditional CV risk factors [9,10]. SARS-CoV-2 infection is associated with systemic inflammation, an altered immune response, and endothelial dysfunction with subintimal inflammation leading to functional and structural arterial remodeling [11,12,13,14,15,16].

Therefore, COVID-19 can have an impact on arterial stiffness. The relationship between increased arterial stiffness and the severity and duration of chronic inflammation in many systemic inflammatory diseases and cardiometabolic syndrome (CMS) is well understood [17,18,19]. Moreover, arterial stiffness is considered an additional CV risk factor, including a risk factor for CV death [12,20].

One method of assessing arterial stiffness is photoplethysmography (PPG) [21,22]. This method has the potential for early screening for CVD risk due to its non-invasive nature, low cost, and mobility of the PPG device. However, data on the significance of parameters that assess arterial stiffness in patients after COVID-19 are scarce [12,20,23,24,25]. Only a continuous increase in the baseline heart rate (HR) is associated with a higher risk of CVD [26].

The present study aimed to investigate whether and to what extent arterial stiffness measured by PPG differs in Polish convalescents after COVID-19 in terms of both the severity of the acute phase of the disease and the time elapsed since its onset.

## 2. Materials and Methods

### 2.1. Study Group

Volunteers for the study were recruited among patients after COVID-19 who were admitted to the Cardiac Rehabilitation Department of the Ustron Health Resort (Poland) for rehabilitation. All consecutive COVID-19 convalescents who gave their written consent to the study were included. The recruitment process lasted six months (from August 2021 to January 2022), and 248 people qualified for the analysis of arterial stiffness. For 23 participants, no results of reproducible measurement of arterial stiffness could be obtained, and these data were excluded from further analysis. Eventually, data from 225 patients were analyzed.

In all patients included in the analysis, the diagnosis of COVID-19 was based on reverse transcription polymerase chain reaction testing or the qualitative assessment of the presence of the SARS-CoV-2 antigen in nasopharyngeal swabs. Patients were divided into subgroups according to sex (102 men and 123 women) and the severity of the acute period of COVID-19, i.e., according to four stages of the disease (stage I—mild (*n* = 112), stage II—moderate (*n* = 58), stage III—severe (*n* = 35), and stage IV—critical (*n* = 15)) based on retrospective data from hospital records and according to the guidelines of the Polish Society of Epidemiologists and Infectiologists [27]. In addition, three subgroups of patients were also distinguished based on the time which elapsed since the disease onset, i.e., <12 weeks (*n* = 24), 12–24 weeks (*n* = 83, and >24 weeks (*n* = 95). The long COVID-19 syndrome was defined as signs and symptoms that continue or develop after acute COVID-19 and includes both ongoing symptomatic COVID-19 (from 4 to 12 weeks) and post-COVID-19 syndrome (12 weeks or more) [28].

The research was approved by the Bioethical Committee of the Medical University of Silesia in Katowice, Poland (Resolution no. PCN/CBN/0052/KB1/68/1/21/22).

### 2.2. Arterial Stiffness Measurement

The analysis of the pulse wave shape obtained through photoplethysmography using the Pulse Trace PCA2 device (Micro Medical Ltd., Rochester, Kent, UK) was used to measure arterial stiffness. The measurement was preceded by acclimatization in the supine position for ten minutes in a quiet room at room temperature. Next, the measurement was carried out in the supine position at the level of the phalangeal artery. Each measurement lasted ten seconds. The measurements were repeated five times for each patient.

The typical digital volume pulse (DVP) contour was analyzed as follows: (1) the first part of the DVP (the so-called systolic component) is caused by the propagation of the pulse wave from the aorta to the finger; (2) the second DVP component (the so-called diastolic component) illustrates the sum of the pulse waves reflected from the small arterioles (known as resistance arterioles); this wave returns to the aorta, and from there it propagates again towards the finger.

Based on the pulse wave contour analysis, the following parameters were automatically calculated: stiffness index (SI), reflection index (RI), and HR. The SI (m/s) measures large artery stiffness and is calculated as the subject’s height divided by the distance between the first systolic peak and the reflected peak. The RI (%) measures the vascular tone of small arteries and is calculated using the a/b × 100% formula, where “a” is the reflected peak, and “b” is the early systolic peak.

The SI and RI parameters depend on the elasticity of the arteries. The lower the elasticity, the higher the SI and RI values because in stiff arteries, the speed of propagation of the pulse wave increases.

### 2.3. Statistical Analysis

Statistical analyses were performed using Statistica 13 (StatSoft; Statistica, Tulsa, OK, USA). The normality of the distribution of the variables was assessed using the Shapiro–Wilk test. The continuous variables were expressed as mean (M) and standard deviation (SD), while categorical variables were shown as absolute numbers (*n*) and percentages (%). Spearman’s rank correlation coefficient, r, was computed to assess the relationships between the variables. The categorical variables between the analyzed subgroups of patients were compared with chi-square test. We used a one-way variance ANOVA to evaluate analyzed subgroups by sex. The combined effects of sex and the severity of COVID-19 (in subgroups according to the severity of the disease, i.e., stages I–IV) as well as the possible changes in RI, SI, and HR values depending on the time since the disease onset were analyzed using a two-way factorial ANOVA. Multiple linear regression was used to analyze the impact of adjusting factors, i.e., sex, age, body mass index (BMI), the severity of the acute COVID-19, the time from the disease onset, smoking status, hypertension, and diabetes on arterial stiffness. Results were considered as significant when *p* < 0.05.

## 3. Results

### 3.1. Characteristics of the Study Group

The study involved 225 convalescents with COVID-19 at the age of 58.98 ± 8.57 years. Of these, 123 (54.7%) were women. The mean time from COVID-19 diagnosis to study enrollment was 24.76 ± 11.79 weeks. Table 1 demonstrates the characteristics of the entire COVID-19 convalescent group as well as subgroups depending on sex.

The mean age and the mean BMI were similar between women and men. There were no differences in diabetes, dyslipidemia, and hyperuricemia prevalence between men and women. However, men had higher mean systolic and diastolic blood pressure (BP) and a significantly higher percentage of diagnosed hypertension than women. Furthermore, we observed that both smokers and former smokers are more often male than female (Table 1). In the group of smokers, almost 81% of the analyzed patients were former smokers who had not smoked for an average time of 20 years. In the study population, mean values of both SI and RI were higher in men than in women (*p* < 0.001). However, HR was similar in both groups (*p* = 0.286). A strong positive correlation between the SI and RI values (r = 0.569, *p* < 0.001) was observed in the analyzed group of COVID-19 convalescent participants—the greater the mean value of RI, the greater the value of SI on average. Similar correlations between SI and RI were observed in both sex subgroups (r = 0.464, *p* < 0.001 for women and r = 0.564, *p* < 0.001 for men). In addition, there was a weak negative correlation between RI and HR (r = −0.302, *p* < 0.001) for the entire group. No correlation was found between SI and HR.

### 3.2. Analysis of Stiffness Parameters Depending on the Severity of COVID-19 Disease

There were no differences in the mean values of arterial stiffness parameters depending on the severity of the acute COVID-19 period. In addition, no significant differences in the distribution of smokers, hypertensive subjects, and patients with diabetes were found depending on the severity of the disease (Table 2).

**Figure 1:**
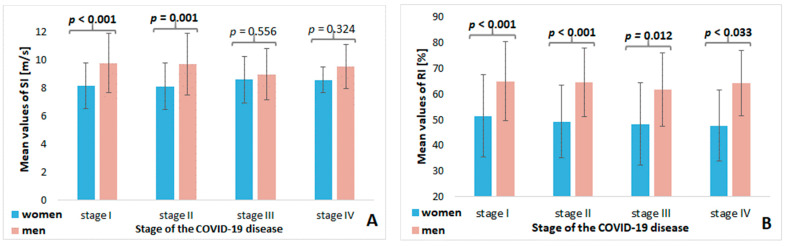

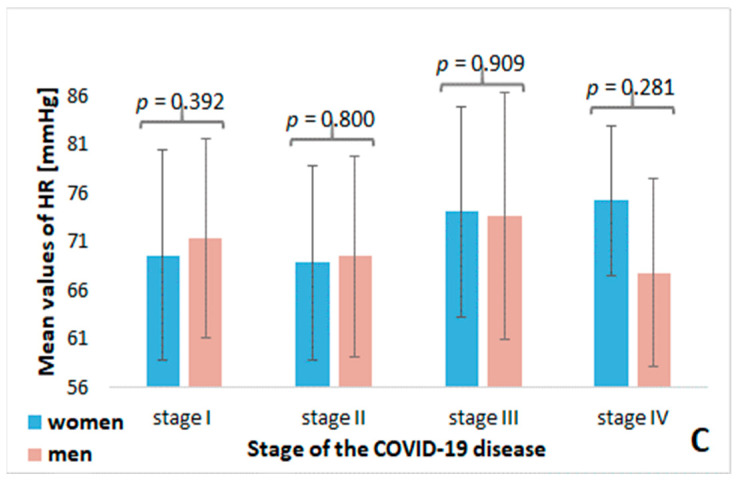
Differences in the parameters of SI, RI, and HR in the specific stages of acute COVID-19 based on sex ((**A**)—differences in SI parameter; (**B**)—differences in RI parameter; (**C**)—differences in HR). SI—stiffness index; RI—reflection index; HR—heart rate; Statistical differences are in bold.

However, significant differences in SI values between women and men were observed only with less severe acute disease (stage I and II), with no differences in the SI values in stages III and IV of COVID-19 (Figure 1A). The RI parameter differed in convalescents of both sexes, regardless of the severity of acute COVID-19 (Figure 1B). There were no significant differences in the respondents’ HR depending on the disease stages (Figure 1C).

### 3.3. Analysis of Stiffness Parameters Depending on the Time from the Disease Onset

Table 3 presents the characteristics of the variables depending on the time from the onset of COVID-19 disease. In the entire study group, the mean values of SI and RI parameters did not differ depending on the time from the acute phase of COVID-19 (*p* = 0.472 and *p* = 0.321, respectively). On the contrary, the mean value of HR was the highest in patients who suffered COVID-19 less than 12 weeks before the study, while the mean HR was the lowest in patients who suffered COVID-19 more than 24 weeks before; the difference was significant (*p* = 0.002).

**Figure 2:**
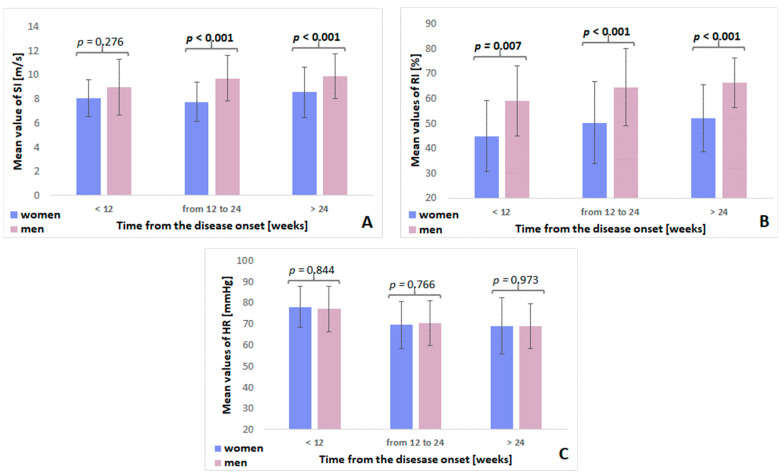
Differences in the parameters of SI, RI, and HR in the specific stages of the onset of COVID-19 based on sex ((**A**)—differences in SI parameter; (**B**)—differences in RI parameter; (**C**)—differences in HR). Significant differences are in bold.

However, significant differences in the mean values of these parameters depending on sex were observed (Figure 2).

There was no difference in the mean SI values between women and men in the period of up to 12 weeks from COVID-19 (*p* = 0.276). However, the differences were statistically significant (*p* < 0.001, each) for the periods of 12–24 weeks and over 24 weeks after the disease (Figure 2A).

The mean RI values differed significantly between the women’s and men’s groups for each period from the onset of the disease (i.e., up to 12 weeks (*p* = 0.007), 12 to 24 weeks (*p* < 0.001), and after 24 weeks (*p* < 0.001)) (Figure 2B).

There were no significant differences in the mean HR between women and men in any of the time intervals analyzed (Figure 2C).

### 3.4. Multiple Linear Regression Analysis of the Influence of Selected Risk Factors for Arterial Stiffness

Multiple linear regression after adjusting for factors influencing arterial stiffness, i.e., sex, age, BMI, the severity of the acute phase of COVID-19, the time from the disease onset, smoking status, hypertension, and diabetes confirmed that age, sex, time from disease onset as well as diabetes are independent determinants that could influence arterial stiffness. The exact results from the analysis are presented in Table 4.

## 4. Discussion

In the analyzed population of 225 COVID-19 convalescent participants, the mean values of RI and HR depended on the time elapsed since the onset of infection. Patients over 24 weeks after COVID-19 had lower mean values of HR than patients below 12 weeks from the disease. On contrary, patients below 12 weeks from the disease had lower RI than patients more than 24 weeks from the disease. In addition, the mean values of SI, and RI were influenced by age, sex, and diabetes. The influence of the above-mentioned parameters on arterial stiffness was independent of other cofactors, such as body mass index, smoking, and hypertension. There were no differences in SI, RI, and HR in terms of the severity of acute COVID-19 symptoms. The mean values of SI and RI were higher for men than women, while HR was similar for both sexes.

Arterial stiffness gradually increases with age [26,29,30]. In our study, age influenced arterial stiffness only via the SI parameter. This may be because our study group was homogenous in case of age. Only 14% of the analyzed patients were under the age of 50, while only 6% of patients were over 70 years old. Moreover, arterial stiffness parameters have been shown to differ between men and women, which may be related to the effect of estrogens on the vascular wall [26,31,32]. It has been documented that arterial stiffness increases significantly in post-menopausal women [33]. Among other factors influencing arterial stiffness, the following ones can be distinguished: hypertension, increased pulse, diabetes, dyslipidemia, insulin resistance, chronic kidney disease, smoking, excessive alcohol consumption, infections as well as lack of physical activity. In our study, the mean values of the parameters describing arterial stiffness (i.e., SI and RI) were higher in men. Although most women in the present study were in the peri- or post-menopausal period, some protective effects of estrogens on the vascular wall cannot be ruled out [31]. In turn, no differences in terms of age and BMI were observed between the sexes. Also, the mean BP values and the percentage of smokers and former smokers were higher in the group of men than in the group of women. This may be one of the explanations for the differences in RI and SI values between the sexes, as increased systolic BP and smoking status were associated with increased vascular tone and the progression of atherosclerosis [34,35,36]. In our analysis, strong positive correlations between the SI and RI values in the entire analyzed group of COVID-19 convalescents as well as in sex subgroups were demonstrated. An earlier study by Madhura and Sandhya [37] also showed a positive correlation between the two parameters.

An interesting observation from the present study is a correlation between RI and HR, and the time that has elapsed since the beginning of the disease, however, in an opposite way. The positive correlation between time from the disease and the RI parameter may reflect the chronic inflammation with progressive arterial wall remodeling, and thus a persistent increase in cardiovascular risk in the convalescents [11,12,13,14,15,16,17,18,19,24,38,39,40]. On the other hand, the inverse correlation of HR with time from disease onset may reflect a resolution of pulmonary lesions with an improvement in lung function and a decrease in dyspnea. The impact of COVID-19 on the increase in arterial stiffness has been observed both in the acute period of the disease [20,41,42] and in convalescents up to 12 months after recovery [40,43]. The acute phase of COVID-19 is dominated by microvascular dysfunction, related to inflammation and oxidative stress, and mediated by an increase in pro-inflammatory cytokine production and diminished nitric oxide bioavailability [15,24]. In the chronic phase, vascular wall remodeling may progress due to persistent endothelial dysfunction, chronic subintimal inflammation, a loss of elastic fibers, and an increase in the inelastic collagenous components of the vascular wall, leading to accelerated vascular aging [15,39,40].

Resting HR is an easy and non-invasive vital sign related to CVD. Previously, it was confirmed that a higher resting HR is associated with increased arterial stiffness [35]. Analyzing differences in HR values regarding the time elapsed since the onset of COVID-19, we observed that HR was lower in patients over 24 weeks from the disease compared to those below 12 weeks. Such a relationship was similar in both women and men. However, no sex-based differences in HR values were observed between the entire group of patients and the different subgroups in terms of the severity of the acute phase of the disease. No significant differences in HR between men and women were observed by Ring et al. [26], despite significant differences in arterial stiffness based on sex.

Surprisingly, in the present study, we demonstrated that mean values of SI and RI did not differ between the subgroups according to the severity of COVID-19. A previous study by Raisi-Estabragh et al. [44] also demonstrated no correlation between arterial stiffness and COVID-19 status in both univariate and multivariate analyses. In contrast, Kumar et al. [42] reported higher arterial stiffness in patients after severe COVID-19. However, this analysis concerned patients in the acute period of the disease, not convalescents.

The present study has some limitations. First, the analyses were performed on a relatively small number of participants. However, we cannot now enlarge the study group in the rehabilitation center because the Polish public payer (i.e., National Health Fund) has terminated the program for COVID-19 convalescents. Second, the study group is not fully representative because only some convalescents decided to join the NHF program. Third, most of the patients were over 60 years of age; only a few participants under the age of 50 were recruited. Finally, some of the data about the medical history of COVID-19 patients was obtained from a review of medical records. The retrospective nature of this search might mean that certain information is lacking. The last but not least limitation of our study is the method of measurement of arterial stiffness. In the present study, we used photoplethysmography through the finger to evaluate arterial stiffness, while the gold standard is the pulse wave velocity method.

## 5. Conclusions

In the present study, we demonstrated that the arterial stiffness in COVID-19 survivors does not depend on the severity of the infection but it is determined by sex, and the time elapsed since the onset of the disease. Age and diabetes were additional factors that may influence the SI and HR values, respectively, in the population of Polish COVID-19 convalescents.

## Figures and Tables

**Table 1:** Characteristics of the total group of COVID-19 convalescents and subgroups based on sex.

	Total Group *n* = 225	Men *n* = 102 (45.33%)	Women *n* = 123 (54.67%)	*p*
Age (years), M ± SD	58.98 ± 8.57	59.18 ± 8.56	58.81 ± 8.61	0.475
BMI (kg/m^2^), M ± SD	29.35 ± 4.82	29.41 ± 5.09	29.30 ± 5.38	0.752
Smoking status, *n* (%)				**<0.001**
Non-smokers	126 (56.00)	42 (41.18)	84 (68.29)	
Smokers	17 (7.56)	14 (13.72)	3 (2.44)	
Former smokers	81 (36.00)	46 (45.10)	35 (28.45)	
Hypertension, *n* (%)	139 (61.78)	73 (71.57)	66 (53.66)	**0.006**
Diabetes, *n* (%)	50 (22.73)	27 (12.27)	23 (10.45)	0.163
Hyperuricemia, *n* (%)	15 (7.54)	5 (2.51)	10 (5.03)	0.382
Dyslipidemia, *n* (%)	116 (52.73)	58 (26.36)	58 (26.36)	0.140
SI (m/s), M ± SD	8.80 ± 1.95	9.54 ± 2.07	8.19 ± 1.62	**<0.001**
RI (%), M ± SD	56.85 ± 16.28	64.29 ± 14.25	50.68 ± 15.29	**<0.001**
HR (beats/min.), M ± SD	70.35 ± 10.67	70.87 ± 10.77	69.91 ± 10.62	0.286
SP (mmHg), M ± SD	139.93 ± 17.42	141.66 ± 16.96	136.67 ± 17.63	**0.031**
DP (mmHg), M ± SD	83.10 ± 11.02	86.48 ± 11.25	80.30 ± 10.03	**<0.001**

M—mean; SD—standard deviation; SI—stiffness index; RI—reflection index; HR—heart rate; SP— systolic pressure; DP—diastolic pressure. Significant differences are in bold.

**Table 2:** Comparisons between the subgroups of COVID-19 convalescents depending on the severity of the acute phase of the disease (i.e., stage I, stage II, stage III, stage IV).

	Stage I *n* = 112	Stage II *n* = 58	Stage III *n* = 35	Stage IV *n* = 15	*p*
Age (years), M ± SD	58.82 ± 7.93	57.64 ± 8.29	61.14 ± 10.49	59.13 ± 8.93	0.299
Sex (M/F), *n* (%)	52 (46.4)	24 (41.4)	17 (48.6)	7 (46.7)	0.902
BMI (kg/m^2^), M ± SD	29.60 ± 4.76	29.18 ± 5.41	29.95 ± 4.42	26.87 ± 3,61	0.187
Smoking status, *n* (%)					0.736
Non-smokers	59 (53.2)	35 (60.3)	22 (62.9)	8 (53.3)	
Smokers	8 (7.2)	6 (10.3)	1 (2.9)	1 (6.7)	
Former smokers	44 (39.6)	17 (29.3)	12 (34.3)	6 (40.0)	
Hypertension, *n* (%)	69 (61.6)	32 (55.2)	23 (65.7)	13 (86.7)	0.155
Diabetes, *n* (%)	24 (24.1)	12 (20.7)	9 (25.7)	2 (13.3)	0.755
Hyperuricemia, *n* (%)	9 (9.7)	5 (8.9)	1 (2.9)	0 (0.0)	0.378
Dyslipidemia, *n* (%)	67 (59.8)	30 (51.7)	12 (34.3)	6 (40.0)	**0.045**
SI (m/s), M ± SD	8.77 ± 2.01	8.88 ± 2.07	8.84 ± 1.77	9.08 ± 1.36	0.940
RI (%), M ± SD	56.48 ± 16.98	56.59 ± 15.64	56.77 ± 16.18	56.62 ± 15.40	0.999
HR (beats/min.), M ± SD	70.23 ± 10.64	69.12 ± 10.09	73.78 ± 11.87	71.21 ± 9.35	0.221
SP (mmHg), M ± SD	137.33 ± 17.00	139.55 ± 18.58	139.91 ± 14.25	145.13 ± 20.82	0.384
DP (mmHg), M ± SD	82,52 ± 10.60	83.50 ± 11.06	82.29 ± 11.11	86.20 ± 14.50	0.633

M—mean; SD—standard deviation; SI—stiffness index; RI—reflection index; HR—heart rate; SP— systolic pressure; DP—diastolic pressure. Significant differences are in bold.

**Table 3:** Comparisons between the subgroups of COVID-19 convalescents depending on the time from the disease onset (i.e., <12 weeks, 12–24 weeks, and >24 weeks).

	<12 Weeks *n* = 24	12–24 Weeks *n* = 83	>24 Weeks *n* = 95	*p*
Age (years), M ± SD	60.79 ± 11.36	58.86 ± 8.31	57.73 ± 7.61	0.258
Sex (M/F), *n* (%)	12 (50.0)	42 (50.6)	38 (40.0)	0.329
BMI (kg/m^2^), M ± SD	28.06 ± 4.77	29.46 ± 4.48	29.39 ± 4.97	0.418
Smoking status, *n* (%)				0.651
Non-smokers	15 (62.5)	41 (50.0)	57 (60.0)	
Smokers	8 (33.3)	34 (41.5)	31 (32.6)	
Former smokers	1 (4.2)	7 (8.5)	7 (7.4)	
Hypertension, *n* (%)	13 (54.2)	54 (65.1)	56 (58.9)	0.546
Diabetes, *n* (%)	4 (16.7)	23 (27.7)	20 (21.1)	0.414
Hyperuricemia, *n* (%)	2 (8.3)	7 (11.1)	5 (5.3)	0.409
Dyslipidemia, *n* (%)	5 (20.8)	55 (66.3)	47 (49.5)	**<0.001**
SI (m/s), M ± SD	8.52 ± 1.98	8.83 ± 2.02	9.05 ± 1.95	0.321
RI (%), M ± SD	52.48 ± 13.49	58.07 ± 17.30	57.35 ± 15.67	0.472
HR (beats/min.), M ± SD	77.52 ± 11.73	69.94 ± 10.77	68.97 ± 10.05	**0.002**
SP (mmHg), M ± SD	138.71 ± 19.75	137.30 ± 16.61	140,35 ± 17.81	0.514
DP (mmHg), M ± SD	88.04 ± 12.32	81.64 ± 11.50	83.52 ± 10.31	**0.045**

M—mean; SD—standard deviation; SI—stiffness index; RI—reflection index; HR—heart rate; SP— systolic pressure; DP—diastolic pressure. Significant differences are in bold.

**Table 4:** The results of linear regression analysis (uni- and multivariate) of the influence of selected risk factors for arterial stiffness after adjusting age, sex, BMI, stage of the severity of the acute COVID-19, the time from the disease onset, smoking status, hypertension, and diabetes.

Predictors	Univariate			Multivariate		
	Coefficient	Std. Error	*p*	Coefficient	Std. Error	*p*
	SI			SI (R^2^ = 0.155; *p* < 0.001)		
Age (years)	0.050	0.015	**0.003**	0.043	0.014	**0.002**
Sex women vs. men	−0.675	0.123	**<0.001**	−0.667	0.121	**<0.001**
BMI (kg/m^2^)	−0.026	0.027	0.335	NS		
Time from disease onset				NS		
<12 weeks vs. >24 weeks	−0.276	0.288	0.338			
12–24 weeks vs. >24 weeks	0.027	0.210	0.897			
COVID−19 severity				NS		
Stage I vs. stage IV	−0.124	0.215	0.565			
Stage II vs. stage IV	−0.008	0.249	0.973			
Stage III vs. stage IV	−0.056	0.289	0.847			
Diabetes no vs. yes	−0.094	0.157	0.547	NS		
Hypertension no vs. yes	−0.241	0.133	0.072	NS		
Smoking status				NS		
Nonsmoker vs. smoker	−0.477	0.207	**0.022**			
Former smoker vs. smoker	0.062	0.219	**0.778**			
	**RI**			**RI** (R^2^ = 0.218; *p* < 0.001)		
Age (years)	0.060	0.127	0.638	NS		
Sex women vs. men	−6.807	0.993	<**0.001**	−7.285	1.038	**<0.001**
BMI (kg/m^2^)	−0.086	0.226	0.703	NS		
Time from disease onset						
<12 weeks vs. >24 weeks	−3.485	2.340	0.138	−4.670	2.103	**0.027**
12–24 weeks vs. > 24 weeks	2.101	1.704	0.219	NS	NS	NS
COVID−19 severity				NS		
Stage I vs. stage IV	−0.134	1.802	0.940			
Stage II vs. stage IV	−0.020	2.090	0.992			
Stage III vs. stage IV	−0.150	2.427	0.951			
Diabetes no vs. yes	2.012	1.301	0.123	2.722	1.228	**0.028**
Hypertension no vs. yes	−1.048	1.117	0.349	NS		
Smoking status				NS		
Nonsmoker vs. smoker	−5.494	1.702	**0.001**			
Former smoker vs. smoker	−0.528	1.808	**0.771**			
	**HR**			**HR** (R^2^ = 0.086; *p* < 0.001)		
Age (years)	−0.016	0.083	0.846	NS		
Sex women vs. men	0.477	0.716	0.505	NS		
BMI (kg/m^2^)	−0.072	0.148	0.627	NS		
Time from disease onset					s	
<12 weeks vs. >24 weeks	5.380	1.530	**<0.001**	5.686	1.519	**<0.001**
12–24 weeks vs. >24 weeks	−2.206	1.115	**0.049**	−2.378	1.105	**0.033**
COVID−19 severity				NS		
Stage I vs. stage IV	−0.856	1.167	0.464			
Stage II vs. stage IV	−1.965	1.353	0.148			
Stage III vs. stage IV	2.693	1.572	0.088			
Diabetes no vs. yes	−1.727	0.850	**0.043**	−2.064	0.886	**0.021**
Hypertension no vs. yes	−0.782	0.732	0.286	NS		
Smoking status				NS		
Nonsmoker vs. smoker	−1.150	1.146	0.317			
Former smoker vs. smoker	0.084	1.218	0.945			

BMI—body mass index; SI—stiffness index; RI—reflection index; HR—heart rate; NS—non-significant, did not enter the model. Significant differences are in bold.

## Data Availability

The data presented in this study are available on request in the Department of General and Inorganic Chemistry, Faculty of Pharmaceutical Sciences in Sosnowiec, the Medical University of Silesia in Katowice, (Poland). The data are not publicly available due to privacy restrictions.

## References

[1] World Health Organization Coronavirus (COVID-19) Dashboard. https://covid19.who.int.

[2] World Health Organization Weekly Epidemiological Update on COVID-19 as of 9 November 2022. https://www.who.int/publications/m/item/weekly-epidemiological-update-on-COVID-19---9-november-2022.

[3] Huang C., Huang L., Wang Y., Li X., Ren L., Gu X., Kang L., Guo L., Liu M., Zhou X. (2021). 6-month consequences of COVID-19 in patients discharged from hospital: A cohort study. Lancet.

[4] Rass V., Ianosi B.A., Zamarian L., Beer R., Sahanic S., Lindner A., Kofler M., Schiefecker A.J., Mahlknecht P., Heim B. (2022). Factors associated with impaired quality of life three months after being diagnosed with COVID-19. Qual. Life Res..

[5] Bellan M., Soddu D., Balbo P.E., Baricich A., Zeppegno P., Avanzi G.C., Baldon G., Bartolomei G., Battaglia M., Battistini S. (2021). Respiratory and Psychophysical Sequelae Among Patients With COVID-19 Four Months After Hospital Discharge. JAMA Netw. Open.

[6] Matsushita K., Ding N., Kou M., Hu X., Chen M., Gao Y., Honda Y., Zhao D., Dowdy D., Mok Y. (2020). The Relationship of COVID-19 Severity with Cardiovascular Disease and Its Traditional Risk Factors: A Systematic Review and Meta-Analysis. Glob. Heart.

[7] Pająk A., Jankowski P., Zdrojewski T. (2022). The burden of cardiovascular disease risk factors: A current problem. Kardiol. Pol..

[8] Harrison S.L., Buckley B.J.R., Rivera-Caravaca J.M., Zhang J., Lip G.Y.H. (2021). Cardiovascular risk factors, cardiovascular disease, and COVID-19: An umbrella review of systematic reviews. Eur. Heart J. Qual. Care Clin. Outcomes.

[9] Rezel-Potts E., Douiri A., Sun X., Chowienczyk P.J., Shah A.M., Gulliford M.C. (2022). Cardiometabolic outcomes up to 12 months after COVID-19 infection. A matched cohort study in the UK. PLoS Med..

[10] Xie Y., Xu E., Bowe B., Al-Aly Z. (2022). Long-term cardiovascular outcomes of COVID-19. Nat. Med..

[11] Goshua G., Pine A.B., Meizlish M.L., Chang C.-H., Zhang H., Bahel P., Baluha A., Bar N., Bona R.D., Burns A.J. (2020). Endotheliopathy in COVID-19-Associated Coagulopathy: Evidence from a Single-Centre, Cross-Sectional Study. Lancet Haematol..

[12] Badaras I., Laučytė-Cibulskienė A. (2022). Vascular Aging and COVID-19. Angiology.

[13] Guzik T.J., Mohiddin S.A., Dimarco A., Patel V., Savvatis K., Marelli-Berg F.M., Madhur M.S., Tomaszewski M., Maffia P., D’acquisto F. (2020). COVID-19 and the Cardiovascular System: Implications for Risk Assessment, Diagnosis, and Treatment Options. Cardiovasc. Res..

[14] Saeed S., Mancia G. (2021). Arterial stiffness and COVID-19: A bidirectional cause-effect relationship. J. Clin. Hypertens..

[15] Varga Z., Flammer A.J., Steiger P., Andermatt R., Zinkernagel A.S., Mehra M.R., Schuepbach R.A., Ruschitzka F., Moch H. (2020). Endothelial cell infection and endotheliitis in COVID-19. Lancet.

[16] Abassi Z., Higazi A.A.R., Kinaneh S., Armaly Z., Skorecki K., Heyman S.N. (2020). ACE2, COVID-19 Infection, Inflammation, and Coagulopathy: Missing Pieces in the Puzzle. Front. Physiol..

[17] Zanoli L., Briet M., Empana J.P., Cunha P.G., Mäki-Petäjä K.M., Protogerou A.D., Tedgui A., Touyz R.M., Schiffrin E.L., Spronck B. (2020). Association for research into arterial structure, physiology (ARTERY) society, the european society of hypertension (ESH) working group on vascular structure and function, and the european network for non-invasive investigation of large arteries. vascular consequences of inflammation: A position statement from the ESH working group on vascular structure and function and the ARTERY society. J. Hypertens..

[18] Zanoli L., Boutouyrie P., Fatuzzo P., Granata A., Lentini P., Oztürk K., Cappello M., Theocharidou E., Tuttolomondo A., Pinto A. (2017). Inflammation and aortic stiffness: An individual participant data meta-analysis in patients with inflammatory bowel disease. J. Am. Heart Assoc..

[19] Zota I.M., Stătescu C., Sascău R.A., Roca M., Anghel L., Mitu O., Ghiciuc C.M., Boisteanu D., Anghel R., Cozma S.R. (2021). Arterial Stiffness Assessment Using the Arteriograph in Patients with Moderate–Severe OSA and Metabolic Syndrome—A Pilot Study. J. Clin. Med..

[20] Rodilla E., López-Carmona M.D., Cortes X., Cobos-Palacios L., Canales S., Sáez M.C., Campos Escudero S., Rubio-Rivas M., Díez Manglano J., Freire Castro S.J. (2021). Impact of Arterial Stiffness on All-Cause Mortality in Patients Hospitalized with COVID-19 in Spain. Hypertension.

[21] Clarenbach C.F., Stoewhas A.C., van Gestel A.J.R., Latshang T.D., Lo Cascio C.M., Bloch K.E., Kohler M. (2012). Comparison of Photoplethysmographic and Arterial Tonometry-Derived Indices of Arterial Stiffness. Hypertens. Res..

[22] Brillante D.G., O’sullivan A.J., Howes L.G. (2009). Arterial Stiffness Indices in Healthy Volunteers Using Non-Invasive Digital Photoplethysmography. Blood Press..

[23] Schnaubelt S., Oppenauer J., Tihanyi D., Mueller M., Maldonado-Gonzalez E., Zejnilovic S., Haslacher H., Perkmann T., Strassl R., Anders S. (2021). Arterial stiffness in acute COVID-19 and potential associations with clinical outcome. J. Intern. Med..

[24] Ratchford S.M., Stickford J.L., Province V.M., Stute N., Augenreich M.A., Koontz L.K., Bobo L.K., Stickford A.S.L. (2021). Vascular alterations among young adults with SARS-CoV-2. Am. J. Physiol. Heart Circ. Physiol..

[25] Ring M., Eriksson M.J., Fritz T., Nyberg G., Östenson C.G., Krook A., Zierath J.R., Caidahl K. (2015). Influence of Physical Activity and Gender on Arterial Function in Type 2 Diabetes, Normal and Impaired Glucose Tolerance. Diabetes Vasc. Dis. Res..

[26] Noon J.P., Trischuk T.C., Gaucher S.A., Galante S., Scott R.L. (2008). The Effect of Age and Gender on Arterial Stiffness in Healthy Caucasian Canadians. J. Clin. Nurs..

[27] Flisiak R., Horban A., Jaroszewicz J., Kozielewicz D., Mastalerz-Migas A., Owczuk R., Parczewski M., Pawłowska M., Piekarska A., Simon K. (2021). Management of SARS-CoV-2 infection: Recommendations of the Polish Association of Epidemiologists and Infectiologists as of April 26, 2021. Pol. Arch. Intern. Med..

[28] National Institute for Health and Care Excellence (2020). COVID-19 Rapid Guideline: Managing the Long-Term Effects of COVID-19. https://www.nice.org.uk/guidance/ng188.

[29] O′Rourke M.F., Staessen J.A., Vlachopoulos C., Duprez D., Plante G.E. (2002). Clinical Applications of Arterial Stiffness; Definitions and Reference Values. Am. J. Hypertens..

[30] Stamatelopoulos K.S., Armeni E., Georgiopoulos G., Kazani M., Kyrkou K., Stellos K., Koliviras A., Alexandrou A., Creatsa M., Papamichael C. (2012). Recently Postmenopausal Women Have the Same Prevalence of Subclinical Carotid Atherosclerosis as Age and Traditional Risk Factor Matched Men. Atherosclerosis.

[31] DuPont J.J., Kenney R.M., Patel A.R., Jaffe I.Z. (2019). Sex Differences in Mechanisms of Arterial Stiffness. Br. J. Pharmacol..

[32] Coutinho T., Borlaug B.A., Pellikka P.A., Turner S.T., Kullo I.J. (2013). Sex Differences in Arterial Stiffness and Ventricular-Arterial Interactions. J. Am. Coll. Cardiol..

[33] Lee H.-Y., Oh B.-H. (2010). Aging and Arterial Stiffness. Circ. J..

[34] Grassi G. (2020). Impact of Heart Rate on Arterial Stiffness: Virtual vs. Real Assessment. J. Hypertens..

[35] Whelton S.P., Blankstein R., Al-Mallah M.H., Lima J.A.C., Bluemke D.A., Hundley W.G., Polak J.F., Blumenthal R.S., Nasir K., Blaha M.J. (2013). Association of Resting Heart Rate with Carotid and Aortic Arterial Stiffness: Multi-Ethnic Study of Atherosclerosis. Hypertension.

[36] Scallan C., Doonan R.J., Daskalopoulou S.S. (2010). The combined effect of hypertension and smoking on arterial stiffness. Clin. Exp. Hypertens..

[37] Madhura M., Sandhya T.A. (2014). Effect of Different Phases of Menstrual Cycle on Reflection Index, Stiffness index and Pulse wave velocity in Healthy subjects. J. Clin. Diagn. Res..

[38] Zota I.M., Stătescu C., Sascău R.A., Roca M., Anghel L., Maștaleru A., Leon-Constantin M.M., Ghiciuc C.M., Cozma S.R., Dima-Cozma L.C. (2022). Acute and Long-Term Consequences of COVID-19 on Arterial Stiffness-A Narrative Review. Life.

[39] Chang R., Mamun A., Dominic A., Le N.-T. (2021). SARS-CoV-2 Mediated Endothelial Dysfunction: The Potential Role of Chronic Oxidative Stress. Front. Physiol..

[40] Lambadiari V., Mitrakou A., Kountouri A., Thymis J., Katogiannis K., Korakas E., Varlamos C., Andreadou I., Tsoumani M., Triantafyllidi H. (2021). Association of COVID-19 with Impaired Endothelial Glycocalyx, Vascular Function and Myocardial Deformation 4 Months after Infection. Eur. J. Heart Fail..

[41] Stamatelopoulos K., Georgiopoulos G., Baker K.F., Tiseo G., Delialis D., Lazaridis C., Barbieri G., Masi S., Vlachogiannis N.I., Sopova K. (2021). Estimated Pulse Wave Velocity Improves Risk Stratification for All-Cause Mortality in Patients with COVID-19. Sci. Rep..

[42] Kumar N., Kumar S., Kumar A., Bhushan D., Kumar A., Kumar A., Singh V., Singh P.K. (2021). The COSEVAST Study Outcome: Evidence of COVID-19 Severity Proportionate to Surge in Arterial Stiffness. Indian J. Crit. Care Med..

[43] Ikonomidis I., Lambadiari V., Mitrakou A., Kountouri A., Katogiannis K., Thymis J., Korakas E., Pavlidis G., Kazakou P., Panagopoulos G. (2022). Myocardial Work and Vascular Dysfunction Are Partially Improved at 12 Months after COVID-19 Infection. Eur. J. Heart Fail..

[44] Raisi-Estabragh Z., McCracken C., Cooper J., Fung K., Paiva J.M., Khanji M.Y., Rauseo E., Biasiolli L., Raman B., Piechnik S.K. (2021). Adverse cardiovascular magnetic resonance phenotypes are associated with greater likelihood of incident coronavirus disease 2019: Findings from the UK Biobank. Aging Clin. Exp. Res..

